# Demographic Attributes of Knowledge, Attitude, Practices, and One Health Perspective Regarding Diarrhea in Pakistan

**DOI:** 10.3389/fpubh.2021.731272

**Published:** 2021-11-18

**Authors:** Ariba Abbasi, Khuram Shahzad, Rana Muhammad Kamran Shabbir, Muhammad Sohail Afzal, Hamza Zahid, Talha Zahid, Haroon Ahmed, Jianping Cao

**Affiliations:** ^1^Department of Biosciences, COMSATS University Islamabad, Islamabad, Pakistan; ^2^Department of Lifesciences, University of Management and Technology, Lahore, Pakistan; ^3^Sandeman Provincial Hospital, Quetta, Pakistan; ^4^Faculty of Pharmacy and Health Sciences, University of Balochistan, Quetta, Pakistan; ^5^Key Laboratory of Parasite and Vector Biology, National Health Commission of the People's Republic of China, Shanghai, China; ^6^National Institute of Parasitic Diseases, Chinese Center for Disease Control and Prevention, Shanghai, China; ^7^The School of Global Health, Chinese Center for Tropical Diseases Research, Shanghai Jiao Tong University School of Medicine, Shanghai, China; ^8^World Health Organization (WHO) Collaborating Centre for Tropical Diseases, Shanghai, China

**Keywords:** knowledge, attitude, practices, bacteria, viruses, pathogens and diarrhea

## Abstract

**Background:** Loose bowels is a clinical sign of gastrointestinal transport channel proteins, channels, and physical and chemical boundaries being harmed, prompting issues of water and electrolyte transport in the intestinal system. It is still considered as a major reason for emergency visits to hospitals in low-middle income countries. Zinc is a suitable treatment along with ORS for diarrhea. KAP surveys are usually conducted to collect information about general or specific topics of a particular population. The objective of this study was to investigate the knowledge, attitude, practices (KAP), and one health perspective regarding diarrhea among the participants from urban and rural populations of Rawalpindi and Islamabad, Pakistan.

**Methods:** Data was collected by conducting a survey among residents of twin cities over a period of 6 months (from July 2020 to December 2020). The questionnaire compromised socio-demographic features and the degree of KAP with respect to diarrhea management and control. One way ANOVA tests were applied to observe the demographic relationship and various factors influencing knowledge, attitude, practices, and one health perspective about diarrhea.

**Results:** A total of 338 subjects participated in the study. Female subjects were in the majority with 63% while the rest were male. A majority of the participants were between 15–25 years of age and 79.6% participants were un-married. The leading ethnic group was Punjabi with 52.7%; the lowest ethnic group were of Sindhi ethnicity with 8.6%. Age has a significant association with respect to knowledge and attitude. Religion has a significant association with respect to knowledge, practices, and one health, while education/qualification has an association with knowledge. The rest of the variables found no association with each other.

**Conclusion:** It is concluded from the recent study that most residents of the twin cities of Pakistan knew about diarrhea and had a good attitude and practices toward it. Age, religion, and education have different roles regarding different diseases in the population of Pakistan. The current study has its limitations as well. Parts of the study were conducted in the capital of Pakistan which is more developed as compared to other areas of Pakistan. It would be better to explore the remote areas of Pakistan where basic amenities of life such as education, wealth, and unemployment are not available. It is important to create more awareness among community members. They should be aware how dangerous these viruses and bacteria can be. Other parts of Pakistan should also be explored for better understanding that will help in making a nationwide health policy.

## Introduction

Diarrhea is characterized as three or more loose or liquid stools passing through each day or more frequently than usual for the person. For the most part, loose bowels is a clinical sign of gastrointestinal transport channel proteins, channels, and physical and chemical boundaries being harmed, prompting issues of water and electrolyte transport in the intestinal system (1). With advancements in technology and the medical field, the mortality rate associated with diarrhea has reduced but it is still considered as a major reason for pediatric emergency visits in hospitals, particularly in low-income countries in Asia and Africa (2). The common cause for diarrhea is a variety of bacteria, viruses, and fungus (3). Due to poor hygiene, infection spreads by infected food or drink or from individual to individual. Diarrhea is a condition that is both preventable and treatable. Diarrhea fluid deficiency has fatal consequences and is the leading cause of malnutrition (4). It causes 1.3 million (M) deaths per year and is also a widespread health problem in the world (5).

The treatment of Diarrheal illness as per the World Health Organization (WHO) is Zinc supplementation along with ORS, which has emerged as a potent approach to treating Diarrhea (6). To meet the challenges of prevention of diarrhea, an effective public health program is needed which should include supplies of safe drinking water, zinc supplementation prevention/early correction of dehydration, ultraviolet purification filter plants, and advice on boiling of water at household level (7). Diarrhea can also be treated by Rehydration with intravenous fluids, Nutrient-rich foods including breast milk, and by giving a nutritious diet to children when they are well (8).

An expert consultation identified several barriers in reducing childhood diarrhea–related mortality (9). These included: the absence of national coordination within ministries and other stakeholders to deliver interventions, insufficient financial resources, inadequate training and support for health workers, poor systems for monitoring and assessing key programmatic indicators, and sporadic availability of key commodities. However, care–seeking behaviors by families, and their belief systems around diarrheal diseases, were not identified as possible barriers, although these are well-described in the literature (10).

WHO and UNICEF launched a comprehensive Diarrhea control plan in 2009 (11). Global Action Plan for Diarrhea has an ambitious goal of ending preventable childhood deaths by 2025, and to achieve that, it provides a set of priorities and interventions to scale-up progress at a country level (12). Integrated Global Action Plan for Diarrhea introduces a cohesive approach to ending preventable Diarrhea deaths. But to reach every child would require scaling-up and targeting of interventions known to prevent and control Diarrhea. Enabling this vision requires coordination and collaboration from child-health-related programs, parents, communities, community health workers (CHWs), civil society, and the private sector. Pakistan follows the WHO guidelines and policies in this regard (13).

A KAP survey is usually conducted to collect information on the knowledge, attitudes, and practices about general and/or specific topics of a particular population. KAP surveys can identify needs, problems, and barriers to help plan and implement interventions (14). It deepens the understanding of commonly known information, attitudes, and factors that influence behavior. KAP studies help in assessing and identifying communication processes and sources important for program implementation and effectiveness. It also helps to set program priorities and make program decisions (11).

There is a scarcity of data regarding the KAPs of diarrhea in the Pakistani community, so the present study was designed to assess demographic attributes of knowledge, attitude, and practices and to establish one health perspective toward diarrhea among residents of Rawalpindi and Islamabad, Pakistan.

## Materials and Methods

### Study Area

The study was carried out in universities of the twin cities: the capital Islamabad and Rawalpindi. The terrain consists of plains and mountains in the metropolitan area of Islamabad and Rawalpindi, whose total area exceeds 1,175 m. In general, three general physiographic zones trend east-northeast. In the mountainous terrain of the Margalla Hills lies the northern part of the metropolitan area. Rawalpindi, famed for its ancient Buddhist heritage, is situated on the Pothohar Plateau.

### Proforma Design, Study Design, Study Sample, and Study Participants

Simple random sampling was considered throughout this study and the university students and adjoining areas within the university were considered in the population, by following the (15), for the Demographic variables such as age, gender are categorical data. To assess the correct sample size formula was used. The margin of error is set to 0.05, the estimate of variance is set at 0.50, and the t-value is fixed at 1.65, which resulted in a 264-sample size. However, to overcome the non-response, initially, we set 369 sample sizes, and 31 did not provide a response. Hence, the final sample size of this study is 338 students. The study was done between July and December 2020. The proforma for data collection was designed after a thorough literature survey (4, 16, 17) and consisted of 57 questions. The questionnaire was divided into five main parts. The first part consists of 13 questions that were about the demography. The second part was about the knowledge, with 24 questions. The third, fourth, and fifth parts were about the attitude, practices, and one health with four, eight, and eight questions, respectively.

### Data Collection and Analysis

The participants were briefed about the survey purposes and written informed consent was collected from the participants. Data was entered into an MS excel spread sheet and a database was established. Statistical evaluation was executed by using Jamovi version 1.6.7. One way ANOVA tests were applied to observe the scores of various factors e.g., knowledge, attitude, practices, and one health across demographic variables regarding diarrhea.

## Results

### Socio-Demography

A total of 338 subjects participated in the study. Most of the participants were female with 63.0%, and the rest were male. Most of the participants (72.2%) were between the ages of 15–25, followed by 26–35, 36–45, and below 15 age groups. Based on ethnic groups, most of the participants were Punjabi with 52.7%, followed by Islamabadis/Islamabadians, Kashmiri, and Sindhi. Of the participants, 96.2% were Muslims while 3.8% were non-Muslims. Based on education, 43.5% participants were undergraduate students followed by graduate and intermediate. A majority (46.4%) of the income group earned below 10,000 Rs followed by participants with incomes above 30,000 Rs. monthly. A majority (70.1%) of participants were of urban origin while 29.9% were from rural backgrounds (Table 1).

**Table 1:** Socio demographic background of the participants.

**Variable**	**Characteristics**	**Participants** **(No)**	**Frequency** **(%)**
Age	15–25	244	72.2
	26–35	73	21.6
	36–45	16	4.7
	Below 15	5	1.5
Gender	Female	213	63.0
	Male	125	37.0
Ethnicity	Balochi	2	0.6
	Gilgiti	5	1.5
	Islamabad territory	69	20.4
	Kashmiri	44	13.0
	Pathan	11	3.3
	Punjabi	178	52.7
	Sindhi	29	8.6
Religion	Muslims	325	96.2
	Non-Muslim	13	3.8
Marital status	Married	69	20.4
	Um married	269	79.6
Qualification	Under Matric	14	4.1
	Matriculation	9	2.7
	Intermediate	22	6.5
	Undergraduate	147	43.5
	Graduate	129	38.2
	MSc	2	0.6
	Ph.D.	13	3.8
	Other	2	0.6
Occupation	Art and Design	1	0.3
	Doctor	6	1.8
	Employee in a company	43	12.7
	Engineer	14	4.1
	Housewife	1	0.3
	Lab worker	13	3.8
	Marketing	1	0.3
	Nothing	1	0.3
	Officer	9	2.7
	Other	10	3.0
	Software engineering	2	0.6
	Still a student	235	69.5
	Businessman	1	0.3
	Housewife	1	0.3
Residence	Rural	101	29.9
	Urban	237	70.1
Income per month	Below 10,000 (may include pocket money)	157	46.4
	11,000–20,000	47	13.9
	21,000–30,000	42	12.4
	Above 30,000	92	27.2
No. of family members	<5	78	23.1
	5–7	193	57.1
	7–9	62	18.3
	More than 10	5	1.5
Time spends with animals (Hours per day)	0	203	60.1
	3–5	120	35.5
	6–10	13	3.8
	11–15	2	0.6
Approximate No. of animals owned	0	196	58.0
	<5	99	29.3
	6–10	36	10.7
	11–15	3	0.9
	More than 15	4	1.2
Presence of animal health care facility in the area	Yes	200	59.2
	No	46	13.6
	May be	92	27.2

### Knowledge

Regarding knowledge about diarrhea, 75.1% of the participants knew that microorganisms were a cause of diarrhea. 52.7% of the participants knew about the microorganisms which were the cause of diarrhea and 43.5% of the respondents were aware that diarrhea is caused due to the rotavirus/norovirus or campylobacter. 50.3% of the participants knew what gastroenteritis is while 40.8% of the participants thought that gastroenteritis was an inflammation of the stomach and small intestine; 11.8% thought it was swelling of the stomach and 47.3% had no idea about the definition of gastroenteritis. 60.9% of the respondents were aware about the early symptoms of the disease while 9.5% were unaware. 69.8% of the subjects considered diarrhea to be a dangerous disease, while 47% considered it a disease that lasted for 1 week. 73% think that babies can have diarrhea, 45.3% considered weight loss to be a result of diarrhea, and 41.4% considered lethargy to be a symptom. 71.6% agreed that animals can also get diarrhea, 58.3% think that animal feces could be responsible for spreading diarrhea, and 64.2% believed that diarrhea can cause bleeding in organisms. 71.6% believed that diarrhea affects blood pressure, 92.6% responded that it may be responsible for dehydration, 54.1% believed that diarrhea can affect the baby during pregnancy, 56.8% believe in death due to this, and 55.3% considered ORS as an effective treatment for this disease. 41.4% agreed that a vaccine is available for this disease, 59.6% believed that undercooked food is responsible for diarrhea, and 83.7% believe contaminated water is a source of transmission of disease. 84.6% of the participants considered contaminated food and water a risk factor of diarrhea; 4.1% did not consider them as a risk factor and 11.2% were not sure about it. 48.2% of the participants had suffered with diarrhea while the rest of the participants had not suffered with this disease (Table 2).

**Table 2:** Knowledge toward diarrhea of participants in the study.

**Variable**	**Characteristics**	**Participants** **(No)**	**Frequency** **(%)**
Do you think diarrhea is an infection caused by microorganisms	Yes	254	75.1
	No	10	3.0
	May be	56	16.6
	No idea	18	5.3
Do you know about viruses and bacteria that cause diarrhea?	Yes	178	52.7
	No	111	32.8
	May be	49	14.5
Do you know Rotaviruses /Noroviruses/ Campylobacter are also a cause of diarrhea?	Yes	147	43.5
	No	70	20.7
	May be	74	21.9
	No idea	47	13.9
Do you know what gastroenteritis is	Yes	170	50.3
	No	60	17.7
	May be	55	16.3
	No idea	53	15.7
Gastroenteritis is?	Inflammation of stomach and small intestine.	138	40.8
	Swelling of stomach and small intestine.	40	11.8
	No idea	160	47.3
Do you know about the early symptoms of diarrhea	Yes	206	60.9
	No	32	9.5
	May be	94	27.8
	No idea	6	1.8
Do you think diarrhea is a dangerous disease	Yes	236	69.8
	No	19	5.6
	May be	82	24.3
	No idea	1	0.3
How many days Diarrhea lasts	1 week	159	47.0
	1–4 days	111	32.8
	2–3 weeks	19	5.6
	No idea	49	14.5
Do you think can babies have diarrhea	Yes	247	73.1
	No	16	4.7
	May be	59	17.5
	No idea	16	4.7
Consequences of diarrhea	Lethargy	140	41.4
	Loss of weight	153	45.3
	Unconsciousness	32	9.5
	Death	13	3.8
What do you think animals can get diarrhea?	Yes	242	71.6
	No	27	8.0
	Don't know	69	20.4
Do you think animal feces can spread diarrhea	Yes	197	58.3
	No	22	6.5
	May be	66	19.5
	No idea	53	15.7
Can diarrhea cause bleeding	Yes	217	64.2
	No	38	11.2
	May be	54	16.0
	No idea	29	8.6
Can diarrhea affect blood pressure	Yes	242	71.6
	No	42	12.4
	No idea	54	16.0
Diarrhea can cause dehydration	Yes	313	92.6
	No	11	3.3
	No idea	14	4.1
Can diarrhea affect baby in pregnancy?	Yes	183	54.1
	No	16	4.7
	May be	100	29.6
	No idea	39	11.5
Can diarrhea cause death	Yes	192	56.8
	No	45	13.3
	May be	79	23.4
	No idea	22	6.5
Do you think ORS can be effective treatment for diarrhea?	Yes	187	55.3
	No	9	2.7
	May be	124	36.7
	No idea	18	5.3
Do you think vaccine/medicine for viruses/bacteria which cause diarrhea is available?	Yes	140	41.4
	No	20	5.9
	May be	94	27.8
	No idea	84	24.9
Can eating undercooked meat give you diarrhea?	Yes	201	59.5
	No	30	8.9
	May be	87	25.7
	No idea	20	5.9
Do you think diarrhea can be transmitted through contaminated water?	Yes	283	83.7
	No	20	5.9
	No idea	35	10.4
Is contaminated food and water consumption, a risk factor of diarrhea?	Yes	286	84.6
	No	14	4.1
	May be	38	11.2
Do you ever get infected with this disease?	Yes	163	48.2
	No	175	51.8
Does your family member get infected with this disease?	Yes	175	51.8
	No	123	36.4
	May be	40	11.8

### Attitude

With respect to attitude questions, 84.6% of the participants considered visiting the doctor if they had diarrhea while 4.1% said they would not visit doctor and 11.2% were not sure about visiting. 82.5% believed that avoiding dipping their hand in a vessel could prevent diarrhea. 75.1% believed that use of filtered or boiled water should be used for consumption to avoid diarrhea and 35.2% believed that it can be transmitted form one person to another; 30.8% do not believe this and the rest of the respondents had no idea or were not sure about this (Table 3).

**Table 3:** Attitude toward diarrhea of participants in the study.

**Variable**	**Characteristics**	**Participants (No)**	**Frequency (%)**
Will you visit doctor if you are having diarrhea?	Yes	286	84.6
	No	14	4.1
	May be	38	11.2
Avoiding dipping hand in vessel	Yes	279	82.5
	No	18	5.3
	Sometimes	41	12.1
Filtering/boiling drinking water before use	Yes	254	75.1
	No	22	6.5
	Sometimes	62	18.3
Diarrhea can be transmitted from person-to-person	Yes	119	35.2
	No	104	30.8
	May be	67	19.8
	No idea	48	14.2

### Practices

Regarding practices, 61.8% avoided the formation of ice from tap water while 7.4% had not used tap water for ice and 30.8% sometimes used tap water for ice at home. 87.9% considered that closing the lids of drinking water sources can prevent diarrhea. 86.7% of the participants washed their hands before cooking, eating, or after defecation, while 3.0% of responses were negative and 10.4% sometime washed their hands. 78.4% of the participants mentioned regular cleaning of drinking water vessels while 5.6% said they did not clean them regularly and 16.0% cleaned vessels but not always. 90.2% of the subjects mentioned washing fruits and vegetables before use while 1.2% thought it was not necessary to wash them and 8.6% of participants sometimes washed fruits and vegetables before use. 88.2% wash their hands before feeding their children while 9.5% practice this sometimes. 81.4% practice the proper disposal of refuse material while 74% respondents visit the doctor immediately when they encounter diarrhea (Table 4).

**Table 4:** Practices toward diarrhea of participants in the study.

**Variable**	**Characteristics**	**Participants** **(No)**	**Frequency** **(%)**
Closing the lids of drinking water source	Yes	297	87.9
	No	9	2.7
	Sometimes	32	9.5
Do you avoid ice made with tap water?	Yes	209	61.8
	No	25	7.4
	Sometimes	104	30.8
Hand wash before cooking, eating/after defecation	Yes	293	86.7
	No	10	3.0
	Sometimes	35	10.4
Regular cleaning of drinking water vessels	Yes	265	78.4
	No	19	5.6
	Sometimes	54	16.0
Washing fruits and vegetables before use	Yes	305	90.2
	No	4	1.2
	Sometimes	29	8.6
Washing hands before feeding the child	Yes	298	88.2
	No	8	2.4
	Sometimes	32	9.5
Proper disposal of reuse	Yes	275	81.4
	No	19	5.6
	Sometimes	44	13.0
Visits to doctors	Immediately visit	250	74.0
	After 2 days	67	19.8
	Never visits	21	6.2

### One Health

Regarding one health perspective, 85.8% of the participants knew that human health is associated with the environment while 2.1% negated it and 10.4% were not sure about it. 58.3% of the participants knew about zoonosis while 41.7% had no knowledge about it. 55.6% of the participants considered that diarrhea can be transmitted through animals while 10.4% thought it cannot be transmitted through animals and 16.6% were not sure about that. 63.6% of the participants knew about one health while 36.4% participants had no idea about one health. 33.7% of the participants mentioned that risk of diarrhea was associated with a specific age while 43.2% negated the statement and 14.8% were not sure about that. Almost more than 90% believed that there is a need for proper disposal and sewage systems, 94.1% considered that there is need for public awareness regarding this disease, and 94.1% considered that there must be awareness of complications of untreated prolonged infection (Table 5).

**Table 5:** One-health toward diarrhea of participants in the study.

**Variable**	**Characteristics**	**Participants** **(No)**	**Frequency** **(%)**
Do you think the health of humans is associated with the environment?	Yes	290	85.8
	No	07	2.1
	May be	35	10.4
	No idea	06	1.8
Do you think diarrhea can be transmitted through animals?	Yes	188	55.6
	No	35	10.4
	May be	56	16.6
	No idea	59	17.4
Do you know what zoonosis is?	Yes	197	58.3
	No	141	41.7
Do you think, the risk of diarrhea infection is associated with specific age group?	Yes	114	33.7
	No	146	43.2
	May be	50	14.8
	No idea	28	8.3
Do you know about one health?	Yes	215	63.6
	No	123	36.4
Is there any need of proper disposal and sewage systems?	Yes No	305 33	90.2 9.8
Is public awareness necessary to prevent infectious diseases?	Yes No	318 20	94.1 5.9
Should there be awareness of complications of untreated prolonged infection?	Yes No	318 20	94.1 5.9

### Statistical Analysis Using ANOVA

One-way ANOVA was applied to establish the relationship between dependent and independent variables. Six independent variables (age, gender, ethnicity, qualification, religion, and marital status) and four dependent variables (knowledge, attitude, practices, and one health) were taken to check their scores across all dependent and independent variables. Age and ethnicity have significant association with respect to knowledge. Religion has significant association with respect to one health while education/qualification has an association with knowledge. The rest of the variables found no association with each other (Table 6).

**Table 6:** Demographic variables across KAPs and one health using one-ANOVA.

	**Knowledge ± SD**	**Attitude ± SD**	**Practices ± SD**	**One health ± SD**
**Age (in years)**				
Below 15	11.00 ± 4.472	3.20 ± 0.837	7.20 ± 1.304	6.40 ± 1.140
15–25	13.02 ± 5.275	2.73 ± 1.111	6.39 ± 2.093	5.79 ± 1.681
26–35	16.55 ± 5.307	2.86 ± 1.347	6.70 ± 2.222	5.92 ± 1.631
36–45	17.38 ± 4.303	2.94 ± 1.436	6.75 ± 1.949	6.31 ± 1.138
*F*-test (*p*-value)	11.37 (0.001)	0.57 (0.632)	0.69 (0.556)	0.770 (0.512)
**Gender**				
Female	14.27 ± 5.28	2.76 ± 1.14	6.52 ± 1.99	5.91 ± 1.52
Male	13.42 ± 5.76	2.80 ± 1.24	6.43 ± 2.29	5.74 ± 1.84
*F*-test (*p*-value)	1.89 (0.169)	0.08 (0.767)	0.126 (0.722)	0.8118 (0.368)
**Ethnicity**				
Balochi	17.00 ± 5.657	3.00 ± 0.000	7.00 ± 0.000	6.50 ± 0.707
Gilgiti	14.20 ± 4.438	3.20 ± 0.447	7.60 ± 0.894	5.60 ± 1.342
Islamabad territory	13.01 ± 4.891	2.93 ± 0.896	6.88 ± 1.266	5.99 ± 1.345
Kashmiri	12.89 ± 4.696	2.70 ± 0.904	6.64 ± 1.630	5.91 ± 1.235
Pathan	10.64 ± 4.985	2.55 ± 1.128	6.09 ± 2.468	5.82 ± 1.401
Punjabi	14.80 ± 5.786	2.75 ± 1.330	6.33 ± 2.416	5.77 ± 1.868
Sindhi	13.69 ± 5.491	2.66 ± 1.289	6.17 ± 2.269	5.93 ± 1.602
*F*-test (*p*-value)	2.15 (0.047)	0.466 (0.833)	1.035 (0.402)	0.239 (0.963)
**Religion**				
Muslim	13.87 ± 5.491	2.75 ± 1.187	6.44 ± 2.130	5.80 ± 1.642
Non-Muslim	16.08 ± 4.609	3.38 ± 0.650	7.69 ± 0.480	7.00 ± 1.225
*F*-test (*p*-value)	2.03 (0.155)	3.65 (0.057)	4.49 (0.35)	6.75 (0.010)
**Marital status**				
Married	15.07 ± 5.66	2.87 ± 1.21	6.71 ± 2.08	5.78 ± 1.50
Un-married	13.67 ± 5.40	2.75 ± 1.17	6.43 ± 2.11	5.87 ± 1.68
*F*-test (*p*-value)	3.623 (0.058)	0.557 (0.456)	0.991 (0.320)	0.142 (0.707)
**Education/Qualification**				
Graduate	15.57 ± 5.805	2.71 ± 1.330	6.47 ± 2.408	5.74 ± 1.712
Intermediate	11.36 ± 4.424	2.64 ± 0.953	6.55 ± 1.299	5.73 ± 1.352
Matriculation	9.89 ± 3.100	2.89 ± 0.782	7.00 ± 1.000	5.78 ± 0.833
Msc	9.00 ± 0.000	2.00 ± 0.000	7.00 ± 0.000	6.00 ± 0.000
Other	9.50 ± 3.536	2.50 ± 0.707	6.50 ± 0.707	5.50 ± 0.707
Ph.D.	18.69 ± 3.591	2.77 ± 1.787	6.23 ± 2.619	6.38 ± 1.502
Under Matric	10.29 ± 2.585	2.64 ± 1.008	5.86 ± 2.143	5.29 ± 1.939
Undergraduate	13.24 ± 5.067	2.87 ± 1.049	6.54 ± 1.949	5.98 ± 1.657
*F*-test (*p*-value)	6.84 (0.001)	0.40 (0.899)	0.31 (0.948)	0.682 (0.687)

## Discussion

The study has assessed the knowledge, attitude, practices, and one health perspective regarding diarrhea among residents of Rawalpindi/Islamabad (twin cities) of Pakistan. 83.7% of the participants were aware that diarrhea spread through contaminated water. A similar kind of study was reported from Karachi where 17% considered polluted water as a cause of diarrhea (4). One study from India reported that 55% of mothers were aware of causes of diarrhea (16). The current study was conducted among residents of twin cities where the literacy rate is highest in Pakistan. The higher literacy rate could be related to the increased knowledge about diarrhea. The contrary results from the Karachi study could be due to the limitation of the Karachi study, which was only conducted among the mothers of children under the age of 5 years (4).

Lethargy is considered as the main consequence of diarrhea. 41.4% of the participants considered lethargy a consequence of diarrhea. Similar kinds of observations were reported from Karachi; 71% of mothers said that diarrhea causes lethargy (4). Our results also showed that most of the study participants had a favorable attitude regarding diarrhea. The study showed that 55.3% of participants thought that ORS was an effective treatment for diarrhea. The same was observed in the National Family Health Survey which found that only 27% of participants use ORS in management (17). In another study from Pakistan, it was reported that 74% of mothers considered ORS as enough of a treatment for diarrhea (18).

The present study also revealed that 56.8% of the participants considered diarrhea to be a dangerous disease which can lead to the death of a person. The same observation was reported in a study from India where 81.7% people considered diarrhea a dangerous disease which can lead to death (18).

The current study reports that 81.4% of participants disposed of refuse material properly. A similar kind of study was reported from India which illustrates that 30.5% of participants disposed of waste material properly (18). The high rate of properly disposed material could be associated with higher literacy levels and people awareness in the advanced cities of Pakistan, while the low ratio from India is associated with the low literacy level in the study duration that was conducted two decades earlier (18).

According to the present survey, 86.7% of the participants washed their hands before cooking, eating, and after defecation. One study of a similar type showed that only 60% and 30% practiced handwashing after defecation and before handling of food, respectively (16). In another study from Karachi, it was reported that 62% of mothers understand the different preventive strategies such as washing hands and keeping the room and the child clean as far as diarrhea prevention was concerned (4).

Statistical analysis showed that age is associated significantly with knowledge and attitude. This may mean that knowledge and attitudes vary with age. Furthermore, knowledge, practices, and one health were associated with religion significantly. This may mean that religion has a major influence on the current knowledge, practices, and one health. Education was also found to be associated with knowledge. The rest of the parameters have no significant association with one another. Our results were in line with a KAPS study from Pakistan (19) and is contrary to the study by Khan et al. (20), suggesting that every disease has their own demographic attributes.

## Conclusion

It is concluded from this study that most residents of the twin cities of Pakistan knew about diarrhea and had a good attitude and practices toward it. Age, religion, and education have different roles regarding different diseases in the population of Pakistan. The current study has its limitations as well. Parts of the study was conducted in the capital of Pakistan which is more developed as compared to other areas of Pakistan. It would be better to explore the remote areas of Pakistan where basic amenities of life such as education, wealth, and employment are not available. To raise awareness among those people, we should convey our messages by means of social media, seminars, and motivational talks. In addition to all these things, education, basic amenities, and health facilities should be provided across Pakistan.

## Data Availability Statement

The original contributions presented in the study are included in the article/supplementary material, further inquiries can be directed to the corresponding author.

## Ethics Statement

The studies involving human participants were reviewed and approved by ERB of COMSATS University Islamabad under CUI/Bio/ERB/2021/50 approval number. The patients/participants provided their written informed consent to participate in this study.

## Author Contributions

AA collected the data. RS and AA wrote the paper. MA and HZ performed the statistical analysis. The study was designed and supervised by KS and HA. RS, HA, TZ, and JC critically revised the manuscript. All authors contributed to the article and approved the submitted version.

## Funding

This study was supported by the National Natural Science Foundation of China (No. 81772225 to JC), the Laboratory of Parasite and Vector Biology, National Health Commission of People's Republic of China (No. WSBKFKT2017-01 to AA), and the Fifth Round of Three-Year Public Health Action Plan of Shanghai (No. GWV-10.1-XK13 to JC). The funders had no role in the study design, the data collection and analysis, the decision to publish, or the preparation of the manuscript.

## Conflict of Interest

The authors declare that the research was conducted in the absence of any commercial or financial relationships that could be construed as a potential conflict of interest.

## Publisher's Note

All claims expressed in this article are solely those of the authors and do not necessarily represent those of their affiliated organizations, or those of the publisher, the editors and the reviewers. Any product that may be evaluated in this article, or claim that may be made by its manufacturer, is not guaranteed or endorsed by the publisher.
